# Burnout in healthcare professionals

**DOI:** 10.4102/safp.v68i1.6235

**Published:** 2026-03-02

**Authors:** Mmaphefo M. Maluleka, Felicity N. Bulo, Arun Nair, Klaus B. von Pressentin

**Affiliations:** 1 Department of Family Medicine and Primary Healthcare, Faculty of Medicine, Sefako Makgatho Health Sciences University, Pretoria, South Africa; 2 Department of Family Medicine, Faculty of Health Sciences, University of the Free State, Bloemfontein, South Africa; 3 Department of Family Medicine, Robert Mangaliso Sobukwe Hospital, Kimberley, South Africa; 4 Division of Family Medicine, Department of Family, Community and Emergency Care, Faculty of Health Sciences, University of Cape Town, Cape Town, South Africa

**Keywords:** burnout, depression, anxiety, suicide risk, substance use

## Abstract

Burnout is a work-related syndrome recognised by the World Health Organization and included in the International Classification of Diseases 11th revision (ICD-11) as resulting from chronic workplace stress that has not been successfully managed. Characterised by emotional exhaustion, depersonalisation, and a reduced sense of professional accomplishment, it predominantly affects healthcare professionals exposed to sustained emotional and organisational demands at the workplace. Global evidence indicates that nearly half of practising clinicians experience burnout, with higher prevalence in emergency medicine, anaesthesiology, surgical subspecialities, radiology, internal medicine, family medicine and primary healthcare, as well as among registrars and students. Burnout is a syndrome that is closely associated with other mental health conditions, such as depression, anxiety, substance use, and increased suicide risk, while compromising patient safety through elevated rates of diagnostic and medication errors. Contributing factors include excessive workloads, administrative burden, limited autonomy, poor work–life balance, and misalignment of personal and institutional values. Effective management requires a dual focus: individual strategies such as mindfulness, exercise, and stress-management programmes, and organisational reforms including workload optimisation, streamlining electronic documentation, leadership development, and value alignment. When recognised early and addressed comprehensively, burnout is reversible with improved clinician well-being and enhanced patient outcomes. A coordinated response from healthcare institutions, professional councils, and policymakers is essential to safeguard the workforce and sustain high-quality health services. This article aims to provide readers with evidence-based strategies to help themselves or to support a colleague who may be experiencing burnout.

## Introduction

Burnout is recognised by the World Health Organization in the International Classification of Diseases 11th revision (ICD-11) as a ‘syndrome conceptualised as resulting from chronic workplace stress that has not been successfully managed’.^1,2,3,4,5^ It is not classified as a medical condition, but remains a significant occupational health concern. The three core dimensions of burnout are characterised by: emotional exhaustion (feelings of energy depletion), depersonalisation (increased mental distance, negativity, or cynicism towards work), and a reduced sense of personal accomplishment or professional efficacy.^1,2,3,5^ This condition predominantly affects those working in human service professions, including healthcare, where the emotional and psychological demands are high.^2^ This section provides a foundational understanding of the concept, its recognition by global authorities, and the implications of burnout within high-stress professions such as healthcare. By framing burnout not merely as an occupational issue but a systemic challenge, we underscore the urgency of early identification and intervention to protect both workforce and patient care.

## Epidemiology impact

Global research interest in burnout has grown markedly, with PubMed indexing over 33 000 studies, of which more than half were published between 2020 and 2025. National data indicate that nearly half of healthcare providers experience burnout, with higher prevalence noted among urology, emergency medicine, anaesthesiology, surgical subspecialities, radiology, and internal medicine.^1,2,3,4,6^ Burnout is also highly prevalent among medical students, affecting over half of them during their academic journey, and is frequently linked to psychological decline and suicidal thoughts.^7^ Key contributors to this phenomenon include overwhelming academic demands, imbalance between personal and professional life, financial burdens, and intense pressure from continuous assessments and competitive clinical evaluations.^7^ These challenges closely resemble those encountered by registrars in postgraduate training, where extended working hours, substantial clinical responsibilities, and insufficient institutional support foster emotional fatigue and a sense of detachment.^8,9^

Some studies suggest women may be slightly more affected, although large systematic reviews show no definitive gender difference.^2,7,10^

Comparisons between care settings indicate that outpatient providers report more emotional exhaustion than their inpatient counterparts, although depersonalisation and reduced professional accomplishment rates do not differ significantly.^2,3,5,11^ This sharp increase in literature mirrors the heightened awareness within clinical and public health settings, where burnout is now seen as a critical determinant of healthcare workforce sustainability and patient safety. The cumulative evidence reinforces the need for global and local health systems to prioritise burnout prevention and management.^2,5,6^

The impact of burnout extends beyond individual clinicians to patient safety and health system performance. Burnout is linked to depression, anxiety, substance use, and increased suicide risk, particularly when compounded by underlying mental health disorders.^2,12,13^

Clinical consequences include higher rates of errors in medication and diagnosis, inadequate physician–patient rapport, and poorer patient outcomes.^2^ While health systems experience reduced productivity, lower job satisfaction, absenteeism, presenteeism, and higher staff turnover, straining already limited resources and jeopardising Sustainable Development Goal 3 (Good health and well-being).^3,14^

## Contributing factors

### The current stressed healthcare system

In South Africa, the healthcare sector is currently facing severe challenges because of the combined impact of fiscal austerity, societal instability, and a shortage of healthcare personnel.^15^ Over the last decade, budgetary constraints imposed by austerity policies have led to spending reductions and hiring freezes within provincial health departments, limiting the appointment of new staff and delaying the replacement of essential personnel.^15^ These measures have contributed to overcrowded facilities and extended patient waiting times that can contribute to burnout among healthcare professionals.^15^

Healthcare professionals often work in environments where workplace violence, such as verbal or physical aggression from patients or their relatives, is increasingly reported.^16,17^ These incidents erode morale and heighten the emotional burden already created by heavy workloads and limited systemic support, which can lead to or aggravate burnout.^16,17^

Furthermore, factors such as xenophobia not only threaten foreign healthcare workers by creating a hostile and divisive work environment for the entire healthcare workforce but also induce or increase burnout.^18,19^ It also undermines equitable access to care for migrant patients, contradicting South Africa’s constitutional dedication to health as a human right.^18,19^ Although these factors are considered sensitive within the South African socio-political context, these dynamics are crucial for understanding burnout in the South African setting. This denial of care to foreign patients can result in delayed or worsened clinical outcomes, increasing the workload and frustration among healthcare professionals and sustaining the cycle of burnout.^19^

Key risk factors of burnout include long working hours, heavy bureaucratic or administrative tasks, excessive electronic health record use, poor work–life balance, pressure for productivity, and inadequate leadership support.^2,4,13,20^ Additional contributors are a lack of meaningful work, poor collegial support, misalignment of personal and organisational values, poor communication, and limited flexibility or autonomy in scheduling. Sustained high-stress environments, without adequate training in stress management, create a cycle of chronic stress, inadequate coping, absenteeism, and presenteeism, further exacerbating stress and burnout.^2,4,6,20,13^

Understanding these factors not only helps identify at-risk individuals but also informs the design of robust, evidence-based interventions at personal, organisational and policy levels. Addressing these issues comprehensively can disrupt the vicious cycle of stress and burnout and restore professional satisfaction.

## Relationship with depression

Burnout and depression share overlapping features such as fatigue, low energy, and increased negativity, as well as functional impairment. This overlap has led some researchers to view burnout as part of a depression spectrum or as a form of work-related depression.^2,11,12^ Others argue for a distinction, noting that depression can exist independently of occupational stress, whereas burnout is tied to workplace factors.^2,11,12,21^ Importantly, burnout may predispose to depression, and the two can coexist in high-stress environments.

Recognising these nuances is essential for clinicians and policymakers when designing screening tools and interventions to ensure that both conditions are appropriately addressed rather than conflated.^11,12^ A clear conceptual separation supports targeted mental health care while acknowledging overlapping pathways.

## Clinical presentation and assessment

Burnout typically manifests as emotional exhaustion, depersonalisation, and a diminished sense of personal achievement. Signs include declining work quality, increased clinical errors, and disengagement from professional responsibilities, leading to reduced patient satisfaction and early retirement.^2,4,21^ Validated assessment tools include the Maslach Burnout Inventory (MBI), the Copenhagen Burnout Inventory (CBI), the Oldenburg Burnout Inventory (OLBI), the Professional Quality of Life Scale (ProQOL), the Single-Item Burnout Measure, and the Mini-Z survey.^2,4,5,6^ The Maslach Burnout Inventory, specifically the Human Services Survey designed for healthcare professionals, is commonly used and consists of 22 self-report items, each rated on a 0 to 6 scale reflecting how frequently the described feeling occurs. The tool measures burnout across three subscales: emotional exhaustion (9 items, total score 0–54), depersonalisation (5 items, total score 0–30), and personal accomplishment (8 items, total score 0–48). Within this framework, burnout is indicated when a physician’s score is ≥ 27 for emotional exhaustion, ≥ 10 for depersonalisation, and ≤ 33 for personal accomplishment, signifying high burnout in each respective domain.^2,4,5,6,21^

## Preventative strategies

### Primary, secondary and tertiary burnout prevention

Organisations can implement primary (preventive), secondary (early intervention), and tertiary (treatment) strategies (Table 1). *Primary prevention* addresses workplace and system-level causes of chronic stress so that burnout never develops. It will minimise the root causes of burnout by creating a healthy, sustainable working environment. *Secondary prevention* aims to identify early signs of stress and intervene promptly to prevent progression to full burnout. *Tertiary prevention* focuses on the management and rehabilitation of healthcare workers already experiencing burnout, to reduce complications and help them return to meaningful work.^2,13,21^

**TABLE 1:** Summary of preventive measures.

Level	Aim	Typical actions	Examples in healthcare settings
Primary	Prevent burnout onset	Healthy staffing, flexible rosters, electronic health record optimisation and leadership training	Redesign on-call schedules and form peer recognition programmes.
Secondary	Detect and intervene early	Regular screening, stress-management training and early counselling	Quarterly Maslach surveys and rapid referral to employee assistance.
Tertiary	Treat and rehabilitate	Psychological care, return-to-work plans and ongoing peer support	Occupational health-led reintegration and therapy groups.

### The ABC approach to burnout prevention

An additional framework that complements individual and organisational interventions is the ABC approach, a concept grounded in emotional intelligence. This model encourages clinicians to integrate three reinforcing dimensions into their daily practice:

- **A – Awareness:** Recognising personal abilities, performance limits, and stress signals while remaining empathetic towards colleagues and patients.^20,22^
- **B – Balance:** Maintaining equilibrium across family, social, spiritual, and leisure domains, closely linked to sustainable work–life balance.^23^
- **C – Commitment:** Cultivating healthy routines, consistency, and lifestyle choices that support long-term performance and reduce stress.^20,24^

Applying the ABC approach can help clinicians transform insight into actionable habits.

## Management

Enhancing provider wellness and resilience is key to reducing burnout. Resilience, the ability to cope with stress, can be strengthened through practices such as mindfulness, which has been shown to improve stress management.^2,20,25^ Therefore, both personal and organisational strategies are critical. *Individual interventions* include mindfulness, meditation, gratitude journaling, regular exercise, self-care practices, and stress-management programmes, which strengthen resilience and coping capacity.^2,3,4,6,20,25^ Additional *organisational interventions (contextual)* include aligning institutional and individual values, ensuring fair compensation, streamlining electronic documentation, delegating non-clinical tasks, and offering flexible work arrangements such as part-time schedules or remote consultations.^2,3,4,5,26^ The focus must extend beyond crisis response to fostering long-term cultural and structural changes that nurture resilience and well-being at every level of healthcare delivery.

The complexities of the work environment are such that it is usually never ideal, and it is easy to become overwhelmed because of deficiencies and limited resources, leading to burnout and emotional exhaustion.^27,28^ In such settings, employees often adopt the approach of doing the best possible. This can vary between the novice employees and the generational gap with end-of-career employees, where both groups can learn to support each other through mentorship and shared experiences.^29,30,31^

Practical, evidence-informed strategies include:

- **Seeking support:** Burnout thrives in isolation. Intentionally maintaining connections with peers, friends, and family provides a buffer against its effects.^29,30,31^
- **Finding value in work:** Reconnecting with professional purpose and meaning has been shown to improve engagement and resilience.^20,27^
- **Mentorship at work:** Access to an experienced colleague or supervisor for debriefing and guidance strengthens coping skills and mental health.^5,26,29,30,31^
- **Setting boundaries:** Learning to say no and avoiding chronic overload are essential to protect personal energy and prevent burnout.^24^
- **Spreading out leave:** Taking shorter, regular breaks throughout the year supports sustained performance and well-being more effectively than infrequent extended leave.^32^
- **Taking a technology break:** Periodically disconnecting from constant digital demands fosters mindfulness and reduces emotional exhaustion.^20,23^

The South African Depression and Anxiety Group (SADAG) can play a vital supplementary role. The SADAG provides 24 h toll-free crisis helplines, including the Suicide Crisis Helpline (0800 567 567) and the Department of Social Development Substance Abuse Helpline (0800 12 13 14), offering immediate emotional assistance.^33^ It also offers confidential counselling, peer and support groups (over 180 nationwide), referrals to mental-health professionals, psycho-educational resources, and outreach programmes that help in identifying burnout early and in reducing stigma. By integrating institutional wellness programmes with SADAG’s services, healthcare systems can ensure that practitioners have both preventive and reactive support mechanisms.^33^

## Prognosis and call to action

When recognised early and managed through both personal resilience and systemic reforms, provider burnout carries a favourable prognosis, with measurable improvements in clinician well-being and patient care. Conversely, if left unaddressed, burnout may escalate to depression, substance misuse and, in severe cases, suicide.^2^ Given these profound implications for healthcare delivery and outcomes, coordinated action is imperative. Healthcare organisations, regulatory bodies, and policymakers must collaborate to build supportive and sustainable work environments.^2^ Stakeholders, including healthcare leaders, policymakers, and professional councils, are urged to prioritise comprehensive mental-health strategies and to invest in evidence-based wellness and resilience programmes that safeguard both practitioners and the patients they serve.^2^ This call for collective responsibility is echoed globally. The European Commission has put forward recommendations aimed at strengthening the mental health of essential workers,^26^ while the Australian Government has adopted the ‘Every Doctor, Every Setting Framework’, which prioritises the well-being of medical professionals at a national level and addresses systemic and environmental factors impacting the healthcare workforce.^26^ The World Organization of Family Doctors (WONCA) similarly supports initiatives that foster clinician well-being, equitable compensation, and opportunities for purposeful professional growth.^26^

## Conclusion

Burnout is a significant occupational health concern that arises from chronic workplace stress and insufficient coping mechanisms. Healthcare professionals – particularly physicians and nurses – are disproportionately affected. Enhancing awareness and adopting robust preventive strategies are key to reducing its incidence. Early recognition through validated assessment tools, followed by timely intervention, can mitigate complications and improve outcomes. Establishing supportive and adequately resourced workplaces that promote resilience and professional satisfaction is vital for protecting clinicians’ well-being and maintaining safe, high-quality patient care.
